# Hamstring and psoas length of crouch gait in cerebral palsy: a comparison with induced crouch gait in age- and sex-matched controls

**DOI:** 10.1186/1743-0003-10-10

**Published:** 2013-01-30

**Authors:** Tae-Yon Rhie, Ki Hyuk Sung, Moon Seok Park, Kyoung Min Lee, Chin Youb Chung

**Affiliations:** 1 Department of Orthopedic Surgery, Nalgae Hospital, Seoul, South Korea; 2 Department of Orthopedic Surgery, Kwandong University Myongji Hospital, Kyungki, South Korea; 3 Department of Orthopedic Surgery, Seoul National University Bundang Hospital, Kyungki, South Korea

## Abstract

**Background:**

Previous studies have shown that hamstring lengths are often not short in patients with cerebral palsy, which raises concerns over the benefits of distal hamstring lengthening in patients with crouch gait. In this study, the authors measured lengths of hamstrings and psoas muscles in normal subjects mimicking crouch gait and compared these with lengths in cerebral palsy patients with crouch gait.

**Methods:**

Thirty-six patients with cerebral palsy and crouch gait were included in this study, and in addition, 36 age- and sex-matched normal controls were recruited. Hamstring and psoas muscle lengths in patients were evaluated using gait analysis and interactive musculoskeletal modeling software. Muscle lengths were also measured in the normal control group during normal gait and while mimicking crouch gait, and these were compared with those of cerebral palsy patient with crouch gait.

**Results:**

No significant differences were observed between maximum hamstring (p=0.810) and maximum psoas (p=0.456) lengths of patients and controls mimicking crouch gait. However, patients showed significantly shorter excursions of hamstring (p=0.022) and psoas (p=0.036) muscles than controls, whereas no significant excursion differences were observed between controls during normal gait and mimicking crouch gait.

**Conclusions:**

Normal controls mimicking crouch gait and cerebral palsy patients with crouch gait demonstrate similar muscle length patterns. However, mimicked crouch gait did not reproduce the excursion pattern shown by patients with crouch gait, which suggests that reduced hamstring and psoas excursion is an innate characteristic of pathologic crouch gait.

## Background

Cerebral palsy (CP) is a group of disorders of the development of movement and posture, causing activity limitation, that are attributed to non-progressive disturbances that occurred in the developing fetal or infant brain. Musculoskeletal surgery, such as, single event multilevel surgery, is widely performed in cerebral palsy and focuses on improving gait function and pattern. In ambulant patients, three-dimensional gait analysis is usually performed before and after surgery, and is useful for identifying preoperative problems and for evaluating postoperative results by suggesting gait patterns based on dynamic information [1-4]. Crouch gait is defined as the maintenance of excessive knee flexion even when standing erect, and is characterized by excessive hip flexion, knee flexion, and ankle dorsiflexion [5]. Although weaknesses of ankle plantarflexor and knee extensor, stiffness or spasticity of knee flexors, and stiffness or spasticity of hip flexors have been suggested to cause the condition [6,7], the underlying mechanism has not been elucidated.

Delp et al. compared hamstring and psoas lengths in normal controls and in patients with crouch gait using 3-D gait analysis, and found that patient hamstring lengths were similar to or greater than those of controls, and that psoas lengths were shorter in patients [8]. Therefore, they recommended that hamstring lengthening should not be performed in patients with crouch gait. Furthermore, questions have been raised about the need for hamstring lengthening because of the possibility that it could aggravate crouch gait by increasing anterior pelvic tilt and weakening hip joint extension. Hoffinger et al. found that the hamstring lengths during stance phase in most of patients with crouch gait were longer than resting length and that hamstrings functioned as hip extensor during a significant portion of stance phase [9]. Therefore, they suggested that surgeons should be careful to avoid hamstring overlengthening to prevent an increased anterior pelvic tilt and consider lengthening the iliopsoas. On the other hand, surgeons can encounter patients with crouch gait and hamstring contracture, and some orthopedic surgeons still believe that hamstring lengthening is an effective treatment in these patients. In addition, many authors have reported that hamstring lengthening is effective for treating knee flexion contracture and improving joint movement [10-19]. Furthermore, muscle length is not static, and can be affected by the position, that is, muscle lengths in patients with crouch gait and those in normally developing children may not be comparable because body positions differ.

Accordingly, in this study, we sought to identify differences between the pathologic crouch gait of cerebral palsy patients and the mimicked crouch gait of normal controls to better understand crouch gait in patients with cerebral palsy.

## Methods

This study was approved by the institutional review board (IRB protocol number, B-1006-104-002) of Seoul National Universiry Bundang Hospital, and informed consents were obtained from all participants. All investigations were conducted in compliance with the Helsinki Declaration. Thirty-six cerebral palsy patients with crouch gait were included in this study. Inclusion criteria for patients were as follows; 1) spastic diplegia, 2) the ability to walk without an assistive device (GMFCS level I, II), and 3) persistent knee flexion of ≥ 20 degrees. Patients who received gait correcting orthopedic surgery or selective dorsal rhizotomy, and those with any other neuromuscular disease were excluded. In addition, 36 age- and gender-matched normal children without any musculoskeletal disorder were included as controls.

All 72 study subjects underwent a physical examination, which included; flexion, extension, adduction, abduction, internal rotation, external rotation of each joint, the Ely test (a test of rectus femoris contracture), the Silverskiold test (a test of triceps surae contracture), the popliteal angle test (a test of hamstring contracture), and the Thomas and Staheli tests (both tests of hip flexion contracture). Trochanteric prominence angle, thigh-foot angle, and transmalleolar axis were measured to identify torsional deformities of the proximal femur and tibia.

### Acquisition of kinematic data and muscle length measurements

**Figure 1:**
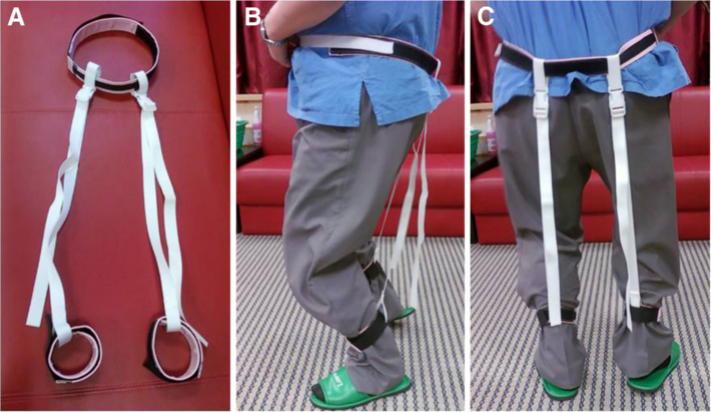
**A: The brace used for inducing crouch gait consisted of a waist belt and two ankle belts with two connecting straps. B**: Lateral view of a normal control wearing the brace **C**: Posterior view of a normal control wearing the brace.

Cerebral palsy patients and normal controls underwent 3-D gait analysis. To induce crouch gait in normal controls, we used a brace designed to limit knee extension. A brace consisted of a waist belt and two ankle belts with two connecting straps, and two straps were tightened to limit knee extension (Figure 1). While mimicking crouch gait, the knee flexion angle of 30 degree was set with a goniometer while standing, and this position was maintained during the stance phase. Mimicked crouch gait was performed at self-selected comfortable walking speed. 3D Gait analysis was performed using a Vicon 370 apparatus (Oxford, United Kingdom) system. Markers were placed for the Helen Meyer marker set by a single experienced operator and 15 reflective markers were used to calculated the kinematic data [20]. Motion was captured while subjects walked barefoot three times on a nine-meter walkway, and the average kinematic data were archived. The spatio-temporal parameters, such as stride length, cadence, and walking speed were also calculated.

**Figure 2:**
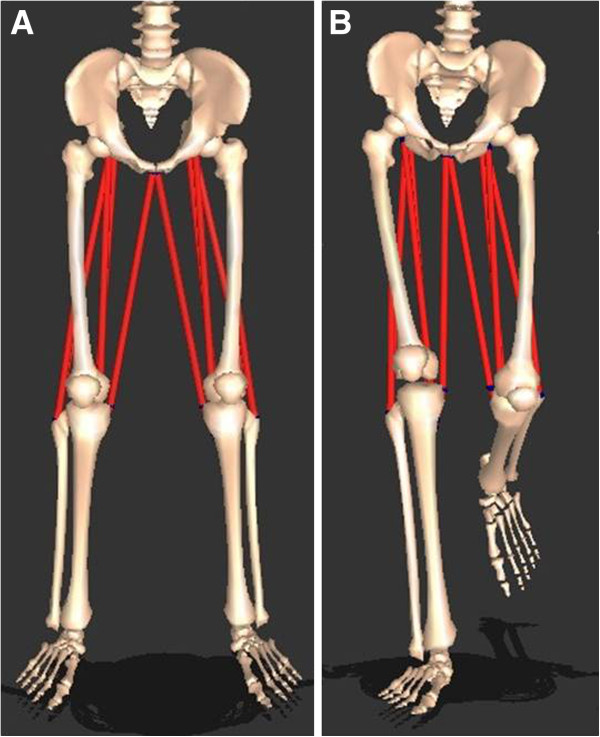
**Three dimensional modeled hamstring lengths were determined using gait kinematic data, and were defined as distances between muscle origins and insertions. A**: Static hamstring lengths were measured when knee and hip joints were in the 0-degree position. **B**: Dynamic hamstring lengths were recorded throughout the gait cycle.

**Figure 3:**
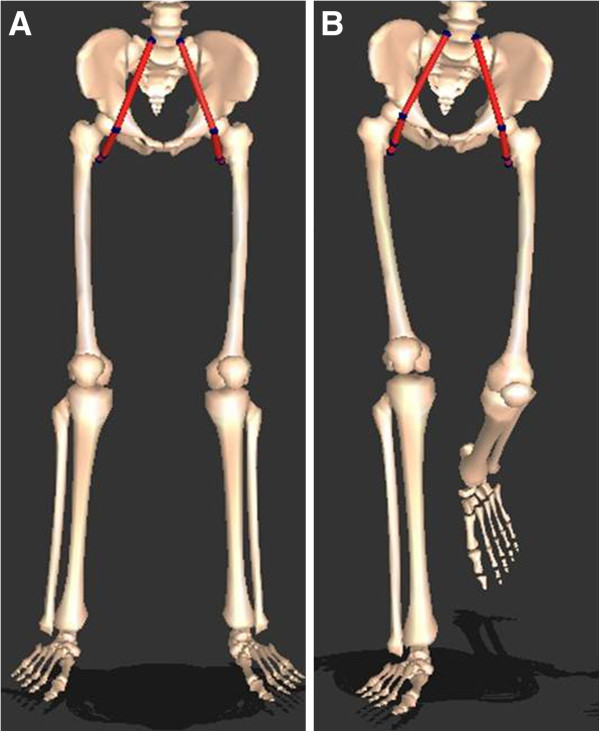
**Three dimensional modeled psoas lengths were obtained using gait kinematic data, and defined as distances between muscle origins and insertions. A**: Static hamstring length was measured when knee and hip joints were in the 0-degree position. **B**: Dynamic hamstring lengths were recorded throughout the gait cycle.

An interactive musculoskeletal modeling program (SIMM, Motion Analysis Corporation, Santa Rosa, CA) was used in conjunction with subjects’ kinematic data to estimated the changes in hamstrings and psoas length during gait cycle. Muscle length was calculated as the distance from origin to insertion of muscles. Absolute and relative lengths of muscles of interest were estimated using this data [21-23]. In this study, we used standardized lengths of hamstring and psoas muscle, which play important roles in crouch gait (Figures 2 and 3). In order to standardize muscle lengths, measured lengths during gait were divided by static muscle lengths in the anatomical position with the knee and hip joint in 0° of extension. Hamstring length was defined as the length of semimembranosus muscle. Hamstring and psoas excursion were calculated by subtracting the minimum muscle length from the maximum muscle length during gait cycle.

Muscle lengths for patients and normal controls with crouch gait and for controls with and without crouch gait were compared.

### Statistics

Normalities of distributions were determined using the Kolmogrov-Smironov test. Mean measures of both lower extremities were used as representative values to ensure statistical independence [24]. The paired *t*-test was used to compare normal controls with and without crouch gait, and to compare patients and normal controls with a crouch gait. The analysis was conducted using SPSS ver. 15.0 (SPSS, Chicago, IL) and p values <0.05 were considered significant.

## Results

Mean age was 16.4±8.9 years (range, 7 to 38 years) in both patient group and normal control group. There were 24 males and 12 females in each group (Table 1).

**Table 1:** Patients demographics and physical examinations

	**Patients**	**Normal controls**	***P***
Number of subjects	36	36	1.0
Age (years)	16.4 (8.9, 7 to 38)	16.4 (8.9, 7 to 38)	1.0
Sex (M:F)	24:12	24:12	1.0
GMFCS level (I/II)	4/32	-	-
Physical examinations			
Thomas test	6.1 (7.7)	0.0 (0.0)	0.003
Popliteal angle (unilateral)	67.1 (13.6)	28.0 (8.4)	<0.001
Popliteal angle (bilateral)	46.1 (16.7)	18.9 (9.0)	<0.001
Ankle dorsiflexion (knee 90° flexion)	21.1 (8.3)	28.1 (10.4)	0.015
Ankle dorsiflexion (knee 90° extension)	7.6 (7.1)	10.0 (8.8)	0.323

Data are presented as mean (SD).

**Figure 4:**
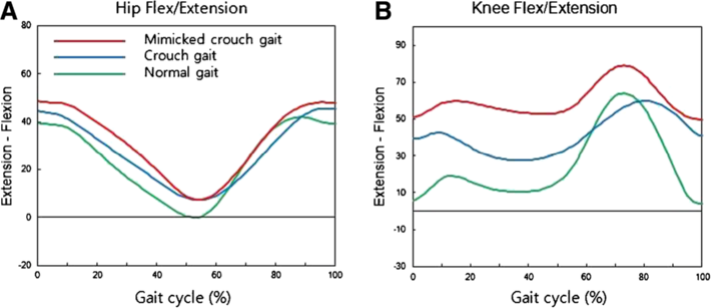
Average sagittal (A) hip and (B) knee angles for mimicked crouch gait, crouch gait and normal gait.

**Figure 5:**
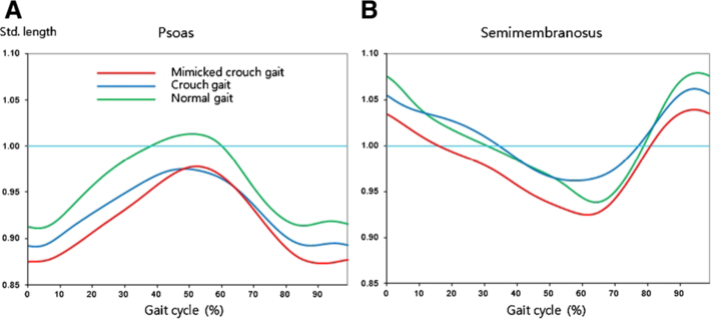
**Standardized (A) psoas and (B) hamstring length during gait cycle for mimicked crouch gait, crouch gait and normal gait.** The length of the semimembranosus was used a s a representative hamstring length.

The minimum hamstring length occurred at maximum knee flexion in swing, and the maximum length during the late swing phase. The minimum psoas length occurred at maximum hip flexion during the late swing phase, and maximum length at minimal hip flexion in stance (Figures 4 and 5).

There was no significant difference in the mean length of semimembranosus muscle between patients with crouch gait and normal controls mimicking crouch gait (p=0.104). The mean length of psoas muscle in patients with crouch gait was significantly longer than that in normal controls mimicking crouch gait (p=0.026). There were no significant differences in the maximum length of hamstring and psoas muscle between patients and normal controls with crouch gait (p=0.810 and 0.456, respectively). Only three patients with crouch gait had a maximum hamstring length that was shorter than controls mimicking crouch gait by more than 1SD. None of patients had a shorter maximum psoas length than controls mimicking crouch gait. Four patients had a maximum hamstring length that was longer than normal controls by more than 1SD. One patient had a maximum psoas length that was longer than normal controls. However, excursions of hamstring muscles and psoas muscles in normal controls mimicking crouch gait were significantly longer than those in cerebral palsy patients with crouch gait (p=0.022 for hamstring muscle and 0.026 for psoas muscle) (Table 2).

**Table 2:** Comparison of muscle lengths and gait parameters between patients with crouch gait and normal controls mimicking crouch gait

	**Cerebral palsy (crouch gait)**	**Control (crouch gait)**	***p***
**Semimembranosus length (%)**			
maximum	105.2 (4.1)	104.9 (3.6)	0.810
minimum	95.1 (3.6)	91.3 (4.6)	0.005
mean	100.1 (3.7)	98.1 (4.0)	0.104
excursion	10.1 (2.2)	13.7 (6.2)	0.022
**Psoas length (%)**			
maximum	98.0 (2.1)	97.3 (3.5)	0.456
minimum	88.8 (1.6)	84.7 (8.0)	0.039
mean	93.4 (1.6)	90.6 (4.9)	0.026
excursion	9.2 (1.7)	12.5 (6.6)	0.036
**Spatio-temporal parameters**			
Cadence (No./min)	95.7 (13.8)	119.7 (14.9)	<0.001
Step length (cm)	38.8 (9.0)	45.6 (121.0)	0.039
Walking speed (cm/s)	63.5 (16.0)	92.8 (19.8)	<0.001
**Kinematic parameters (°)**			
Maximum hip flexion in stance	44.1 (7.1)	50.1 (12.2)	0.066
Minimum hip flexion	7.3 (11.6)	10.4 15.2)	0.473
Hip range of motion	39.3 (10.9)	41.1 (8.5)	0.553
Knee flexion at initial contact	41.6 (13.5)	49.6 (7.3)	0.027
Minimum knee flexion in stance	27.6 (14.2)	47.4 (7.3)	<0.001
Knee range of motion	34.3 (12.5)	33.6 (5.9)	0.836

Data are presented as mean (SD).

There was no significant difference in the mean length of semimembranosus muscle between normal controls mimicking crouch gait and normal controls with normal gait (p=0.095). The mean length of psoas muscle in normal controls mimicking crouch gait was significantly shorter than that in normal controls with normal gait (p<0.001). The maximum length of hamstring and psoas muscle in normal controls mimicking crouch gait were significantly shorter than those in normal controls with normal gait (p=0.013 and <0.001, respectively). However, no significant differences in hamstring (p=0.586) or psoas (p=0.125) excursion were observed between normal controls mimicking crouch gait and normal controls without crouch gait (Table 3).

**Table 3:** Comparison of muscle lengths and gait parameters between normal controls mimicking and not mimicking crouch gait

	**Control (normal gait)**	**Control (crouch gait)**	***p***
**Semimembranosus length (%)**			
maximum	107.3 (1.5)	104.9 (3.6)	0.013
minimum	92.8 (0.9)	91.3 (4.6)	0.160
mean	99.7 (0.9)	98.1 (4.0)	0.095
excursion	14.5 (1.5)	13.7 (6.2)	0.586
**Psoas length (%)**			
maximum	101.6 (0.5)	97.3 (3.5)	<0.001
minimum	91.3 (1.5)	84.7 (8.0)	0.002
mean	96.4 (1.1)	90.6 (4.9)	<0.001
excursion	10.2 (1.3)	12.5 (6.6)	0.125
**Spatio-temporal parameters**			
Cadence (No./min)	120.4 (10.4)	119.7 (14.9)	0.864
Step length (cm)	62.6 (5.3)	45.6 (121.0)	<0.001
Walking speed (cm/s)	126.1 (8.9)	92.8 (19.8)	<0.001
**Kinematic parameters (°)**			
Maximum hip flexion in stance	34.9 (6.3)	50.1 (12.2)	<0.001
Minimum hip flexion	−8.2 (5.7)	10.4 (15.2)	<0.001
Hip range of motion	45.6 (6.1)	41.1 (8.5)	0.071
Knee flexion at initial contact	4.9 (3.7)	49.6 (7.3)	<0.001
Minimum knee flexion in stance	4.5 (3.5)	47.4 (7.3)	<0.001
Knee range of motion	61.1 (5.1)	33.6 (5.9)	<0.001

Data are presented as mean (SD).

## Discussion

In the present study, maximum hamstring and psoas lengths in cerebral palsy patients with crouch gait were similar with those in age- and sex-matched normal controls mimicking crouch gait. However, this study demonstrated that hamstring and psoas excursion in patients were significantly different from those in normal controls.

This study has limitations that should be addressed before we comment on its clinical implications. First, muscle lengths were obtained by measuring lengths from origins to insertions by musculoskeletal modeling and these are likely to be inaccurate in cerebral palsy patients, because musculoskeletal modeling was developed for normal adults and no study has been undertaken to measure its accuracy in pediatric patients or cerebral palsy patients. Second, the brace used in this study was designed to induce crouch gait in normal controls by limiting knee extension. We considered that limited knee extension would create a gait similar to that of cerebral palsy by increasing hip flexion and ankle dorsiflexion by a compensatory mechanism. It could be argued that the physiology of crouch gait and the crouch gait induced in this study are somewhat different. However, we believe that comparisons between these two groups are sounder than those between cerebral palsy patients and normal controls with normal gait, because joint position can affect muscle length. This study shows that hamstring and psoas lengths are dependent on gait pattern, that is, knee joint position. Despite no pathologic muscle contracture in the control group, hamstring and psoas length tended to be shorter in induced crouch gait, and therefore, we believe that comparisons should be made in similar joint positions. Third, this study did not include electromyographic data and gait kinetics. These data could enhance the quality of the results. Therefore, further study on the analysis of electromyographic and kinetic aspect is needed. Fourth, this study did not use the speed-matched control group. Walking speed might be a confounding factor in the analysis regarding muscle length during gait cycle. However, a strength of this study is the age- and gender-matched control group, improving upon previous studies. Further study using age-, gender- and speed-matched control group could enhance the quality of the results. Fifth, the results are valid for patients with GMFCS level I to II spastic CP. These results cannot be generalized to children with lower functional levels or with other clinical sub-types of CP. Sixth, this study did not include the patients with previous gait correcting orthopedic surgery or dorsal rhizotomy. The patients without any previous surgery might have less severe crouch gait than those with a history of surgery. Therefore, our result might be valid only for patients with less muscle contractures.

Several studies investigated the hamstring and/or psoas length in subjects with crouch gait compared with controls with normal gait (Table 4). Delp et al. reported that hamstring lengths in cerebral palsy patients are similar to or longer than those of normal subjects, and that the psoas is shorter than in normal subjects [21]. Since then, the adequacy of hamstring lengthening has been challenged, because it is considered that hamstring lengthening in patients with crouch gait is likely to weaken the flexion force of knee joints and to increase anterior pelvic tilt. van der Krogt compared the peak muscle length between healthy subjects with normal gait and with crouch gait [25]. They also found that the psoas acted at significantly shorter length during the crouch conditions compared to normal, but, the hamstring showed hardly any differences between normal and crouch gait, which concurred with Delp’s study. However, this study showed no difference of maximum length of hamstring and psoas between patients with crouch gait and controls mimicking crouch gait. When comparing normal gait with cerebral palsy patients with crouch gait or induced crouch gait as previous studies, maximum length of hamstring and psoas were sensitive to joint position (Table 3). Therefore, we believe that the results of current study, which compared pathologic crouch gait with non-pathologic crouch gait, is more reasonable than previous studies for demonstrating the cause of crouch gait.

**Table 4:** Previous studies investigating hamstring and/or psoas length in subjects with crouch gait

**Author**	**Case**	**Control**	**Hamstring length**	**Psoas length**
Current study	Crouch gait (32)	Mimicked crouch gait (age-and sex-matched controls, 32)	No difference between two group (p=0.810)	No difference between two group (p=0.456)
Delp (1996) [21]	Crouch gait (14)	Normal gait (10)	Normal or longer length during the crouch gait	Shorter length
Van der Krogt (2007) [25]	Normal subjects with mimicked crouch gait (8)	Normal gait (8)	No difference between two group (p=0.12)	Shorter length during the crouch gait (p<0.05)

Previous studies showed increases in peak hamstring length with increasing walking speed [25,26]. In this study, walking speed in mimicked crouch gait was significantly faster than that in patients with crouch gait (p<0.001). However, there was no difference of peak hamstring and psoas length between two groups. Therefore, if the walking speed in mimicked crouch gait matched that of patients with crouch gait, the peak muscle length in mimicked crouch gait might be shorter than the values in this study. It implied that peak hamstring and psoas length in patients group might be longer than those of speed-matched control group.

There were no significant differences in sagittal gait kinematics of hip joint between patients with crouch gait and normal controls mimicking crouch gait. In terms of sagittal gait kinematics of knee joint, mimicked crouch gait in normal controls was more severe than pathologic crouch gait in patients. In addition, although there were no differences in range of motion of hip and knee joint, the excursion of semembranosus and psoas muscle in mimicked crouch gait were significant longer than those in pathologic crouch gait (Table 2). Therefore, we think these results have meaningful implications. When normal subjects mimicked crouch gait, dynamic muscle lengths seemed to change according to joint position. However, muscle excursion could not be induced in mimicked crouch gait to amount observed in patients, which indicates that decreased muscle excursion in cerebral palsy patients could be regarded as a unique characteristic that cannot be artificially induced. Furthermore, our findings suggest that treatment focus should be placed on increasing excursion. Previous study showed that hamstring excursion was significantly reduced in the short and adequate maximum muscle length groups, and a significant increase in hamstring excursion after botulinum toxin injection was observed only in the short muscle group [3]. We think that surgical intervention such as, distal hamstring lengthening or selective dorsal rhizotomy, might be effective by increasing muscle excursion, not by increasing short muscle length, for treating crouch gait. However, no study has investigated the change of hamstring excursion after surgical intervention. Therefore, further study on these aspect is needed.

## Conclusion

This study demonstrates that normal controls mimicking crouch gait and cerebral palsy patients with crouch gait show similar muscle length patterns. Furthermore, it shows that muscle excursion is a unique characteristic that differentiates cerebral palsy patients with crouch gait and normally developing children mimicking crouch gait.

### Consent

Written informed consents were obtained from all participants for publication of this report and any accompanying images.

## Competing interests

The authors declare that they have no competing interests.

## Authors’ contributions

CYC and MSP have made substantial contributions to conception and design. KML have been involved in acquisition of data, analysis and interpretation of data. TYR and KHS drafted and revise the manuscript. All authors read and approved the manuscript.

## Authors’ information

Tae-Yon Rhie, MD and Ki Hyuk Sung, MD are co-first authors.

This study was supported by Grant No. 03-2010-014 from the SNUBH Research Fund. We have full control of all primary data and we agree to allow the journal to review our data.
